# New indicator of habitat functionality reveals high risk of underestimating trade-offs among sustainable development goals: The case of wild reindeer and hydropower

**DOI:** 10.1007/s13280-022-01824-x

**Published:** 2023-02-09

**Authors:** Martin Dorber, Manuela Panzacchi, Olav Strand, Bram van Moorter

**Affiliations:** 1 Industrial Ecology Programme, Department of Energy and Process Engineering, NTNU, Høgskoleringen 5, 7034 Trondheim, Norway; 2 Norwegian Institute for Nature Research, Høgskoleringen 9, 7034 Trondheim, Norway

**Keywords:** Cumulative impacts, Functional connectivity and ecological networks, Habitat functionality, Land use changes, Renewable energy

## Abstract

Although biodiversity is crucial for Sustainable Development Goals (SDGs), following the current trajectory, we risk failing SDG 15. Using a new indicator quantifying the loss of functional habitat (habitat that is simultaneously suitable and well-connected), we show that the real impact of renewable energy is far larger than previously assumed. Specifically, we estimate that the construction of hydropower reservoirs in south Norway caused a loss of ca. 222 km^2^ of functional habitat for wild reindeer (*Rangifer tarandus*)—which is far larger than assumed based on land inundation indices (110 km^2^). Fully mitigating these impacts is challenging: scenario analyses reveal that the measures proposed by societal actors would yield only a fraction of the habitat lost (2–12 km^2^) and could cause trade-off risks with other SDGs. Using indices of functional connectivity is crucial for environmental impact assessments, as entire ecological networks for several species can be affected far beyond the reservoirs.

**Supplementary Information:**

The online version contains supplementary material available at 10.1007/s13280-022-01824-x.

## Introduction

Sustainable development pathways are required to reconcile human development needs with the protection of the biosphere that humans depend upon (O’Neill et al. 2018). Therefore, biodiversity conservation is crucial for achieving these pathways (Opoku 2019). Sustainable Development Goal (SDG) 14 (Life below water) and 15 (Life on land)—2 of the 17 SDGs developed by the United Nations—are dedicated to protecting biodiversity and ecosystems (UN 2015). Due to the nexus between the goals, the fulfillment of one SDG will lead to positive synergies and negative trade-offs with other SDGs (Liu et al. 2018). Since biodiversity-related SDGs have a positive synergy with almost all SDGs (Blicharska et al. 2019), not reaching SDG 15 involves the risk of triggering complex chains of cascading impacts across all SDGs.

Halfway towards the SDG implementation, 75% of land is significantly altered, and approximately 1 million species are threatened with extinction in the upcoming decades (IPBES 2019), with accelerating trends (WWF 2020). The IPBES (2019) concludes that none of the 11 targets of SDG 15 has a good/positive status or trend when it comes to *“nature and nature’s contributions to people”* (UN 2015). Hence, following the current trajectory, we face the risk of failing the crucial SDG 15 (IPBES 2019).

One possible explanation is that despite having many tools to quantify nexus relationships (e.g., Life cycle assessment, integrated assessment models; Liu et al. 2018), we currently focus on assessing positive synergies but fail to account for negative trade-offs—first and foremost between development and ecological sustainability (Ehrensperger et al. 2019). In other words, current assessments fail to include all relevant sustainability indicators (Lyytimäki et al. 2020).

In addition, current indicators may overly simplistically describe complex chains of causes and effects or may omit background information relevant for correct decision-making (Lyytimäki et al. 2020).

Specifically, although it is well-known that the main cause of the ongoing unprecedented nature decline is the cumulative impact of multiple human drivers (IPBES 2019), and the concept is not novel nor difficult to appreciate, cumulative impacts are very hard to quantify using robust scientific approaches. Cumulative impacts studies belong to a very novel research discipline under rapid development, and, we still lack a widely acknowledged, comprehensive framework to study them, and thus help preventing, monitoring, and mitigating the effects of the rapid piecemeal development of infrastructure causing biodiversity loss.

In this study, we focus on hydropower, which represents the largest source of renewable electricity production (IEA 2021). Land use changes, alteration in freshwater habitat, and in water quality have been identified as the main cause-effect pathways describing the impact of hydropower on biodiversity (Gracey and Verones 2016). Although the impact of hydropower on terrestrial ecosystems can be major (e.g., Pandit and Grumbine 2012; Jones et al. 2016) and does not necessarily correlate with impacts on aquatic ecosystems (Dorber et al. 2020), a larger proportion of literature focuses on freshwater ecosystems (Winemiller et al. 2016; Ashraf et al. 2018; Zarfl et al. 2019; Japoshvili et al. 2021), and in particular on mitigation measures for salmonids, including fish ramps (Haraldstad et al. 2019) or adjustments in minimum flow requirements (Poff et al. 2010).

Here, we focus on the impact of hydropower development in Norway on a terrestrial and migratory species of high conservation value and societal interest: wild reindeer (*Rangifer tarandus tarandus*). The development of infrastructure following the industrial revolution caused significant habitat loss and fragmentation for *Rangifer,* both in Norway (Vistnes and Nellemann 2008; Panzacchi et al. 2013a, b; Panzacchi et al. 2015, 2016; Beyer et al. 2016; Gundersen et al. 2019; van Moorter et al. 2020) and worldwide (Hebblewhite 2017; Joly et al. 2021; Skarin et al. 2015). Several Norwegian valleys have been transformed into hydropower reservoirs, submerging calving areas and migration routes (e.g., Panzacchi et al. 2013a, b). Blocking movement corridors led in several cases to a loss of access to seasonal pastures far from the barrier itself or to fragmented populations (Panzacchi et al. 2016). Habitat loss and fragmentation, among other factors, led to the recent red-listing of the species in the categories “Vulnerable”, globally (Gunn 2016), and “Near Threatened” in Norway (Eldegard et al. 2021). However, until recently, classical ecological approaches did not allow quantifying the real extent of the damage caused by the cumulative impacts of infrastructures such as hydropower.

Here we use a novel, robust indicator focusing on the functionality of the entire reindeer ecological network (van Moorter et al. 2023; van Moorter et al. under revision) to demonstrate how the cumulative impact of hydropower leads to a far stronger negative trade-off between SDG 7 and SDG 15 than previously assumed. Note that we refer to the concept of “habitat functionality” to highlight not only the suitability, but also the effective connectivity (van Moorter et al. 2021) of the landscape for a given species. In other words, functional habitats are at the same time suitable and well connected. Habitat functionality therefore integrates the concept of niche in n-dimensional environmental space, and the real spatial configuration of resources and infrastructures, in geographic space (x–y coordinates)—thus operating in “topological space” (van Moorter et al., under review). The correct estimation therefore requires to consider the cumulative impact of anthropogenic activities simultaneously on habitat loss and habitat fragmentation (van Moorter et al. 2023; van Moorter et al. under revision; see Web App: Panzacchi and van Moorter 2022a).

Storage and pump storage hydropower plants need reservoirs to store water in times of surplus, and to release it with short reaction times, allowing for electricity production during periods of peak energy demand (Egré and Milewski 2002). However, the inundated land area causes both habitat loss (Dorber et al. 2018) and habitat fragmentation for a range of species both in freshwater and terrestrial ecosystems, as the reservoirs can create movement barriers, especially for wide-ranging species such as *Rangifer* (Mahoney and Schaefer 2002). Not only the reservoirs, but the entire network of infrastructure associated with hydropower can severely disrupt ecological networks and reduce functional habitat (e.g., Panzacchi et al. 2013a, b). These include access roads, transformators, power masts, and wide-reaching grids of powerlines that need to be constructed to convey electricity to urban areas. In addition, the presence of reservoirs typically attracts tourism, thus triggering the piecemeal development of infrastructure such as tourist resorts, more roads, hiking trails, and private cottages.

When it comes to sustainability assessment of hydropower and land use change, normally only the impact of the inundated land by reservoirs (quantified as m^2^ land occupation per kWh electricity produced with the related reservoir) and the associated ecosystem quality loss are quantified (Dorber et al. 2020), because this is the value reported in databases, such as Ecoinvent (Wernet et al. 2016) and the Global Reservoir and Dam Database (Lehner et al. 2011). The cumulative impact of renewable energy on biodiversity—which can reach far beyond the reservoirs themselves -, is commonly overlooked or strongly underestimated (e.g., Niebuhr et al. 2022). This may be both because of simplicity, and because of technical and scientific challenges. Due to the recent origin of research on cumulative impact there are currently no widely acknowledged theoretical or methodological frameworks to quantify such impacts in the first place. Consequently, in the absence of quantitative estimates of cumulative impacts it is challenging to agree upon and to implement prevention, mitigation, or offset measures, involving multiple stakeholders (e.g., political, administrative, and legal actors) in multiuse landscapes. The vacuum created by delays in scientific advances, together with the complexity of the problem from a societal and economic perspective, creates the opportunity for inaction, and often leads to failure in adequately handling the trade-offs between conflicting SDG goals. Indeed, one of the targets of SDG 15 aims at restoring ecosystems, which therefore requires efficient mitigation and offset measures (Grimm and Köppel 2019).

This, inter alia, raises the questions: How will the SDG 15 trade-offs risk change if we move from habitat loss to functional habitat loss as an indicator? Can the use of the new indicator lead to new risk management strategies and policy recommendations? Can we mitigate negative trade-offs with SDG 15? If yes, how, and what new trade-off risks can this cause in relation to other SDGs? What role do citizens and stakeholders play in identifying mitigation measures? We answer these questions by focusing on hydropower and wild reindeer in Norway. Norway is one of the top 10 hydropower producers (IEA 2020) and, in the same areas used for energy production, hosts the last remaining populations of wild mountain reindeer in Europe. Norway is now faced with the challenge of revising, re-structuring, and expanding the renewable energy system while improving conditions for the newly red-listed wild reindeer, and ensuring their long-term conservation, nationally and internationally (Bern Convention, Annex III). To balance the trade-off between conservation and development it is crucial that the cumulative impact of hydropower is assessed and included into simulation-based land planning support tools (van Moorter et al. 2023; van Moorter et al., under revision). In this study, we apply the cutting-edge ConScape software (ConScape — Connected Landscapes; van Moorter et al. 2023; van Moorter 2023) to quantify the amount of functional habitat (i.e., high-quality, and well-connected habitat, see van Moorter et al. 2023) currently available to wild reindeer in an area in south Norway. In addition, we estimate the amount of Equivalent Connected Habitat (ECH) [in km^2^] that has been lost due to the construction of hydropower reservoir since 1973. We then compare this estimate to the traditional estimates based on square kilometers of land flooded, or of suitable habitat lost—ignoring connectivity. Finally, in addition to this back-casting exercise, we use the same software to forecast changes in habitat functionality under future scenarios, to assess the efficacy of a list of mitigation or offset measures proposed during an extensive stakeholder participatory process. Last, we discuss new SDG-related trade-off risks that these mitigation measures might cause.

## Materials and methods

### Estimating loss of functional habitat

In several sustainability assessments (e.g., United Nation Environmental Program 2015), the impact of hydropower reservoirs is quantified as the amount of land inundated, which is a very crude estimate. In classical ecological studies, niche modeling approaches are typically used to estimate habitat loss, but typically without accounting for loss of connectivity at the landscape scale. This necessarily underestimates the impacts manifesting beyond the inundated area. Together with a team of ecologists, computer scientists, and network scientists we developed a multistep framework and software (ConScape—Connected Landscapes; van Moorter et al. 2023; van Moorter 2023) to quantify the functionality of the entire ecological network in terms of both habitat quality and connectivity. The procedure integrates niche modeling approaches and network studies, while estimating the cumulative impact of human activities simultaneously on habitat loss and fragmentation. The approach and software have been developed to aid sustainable land planning using reindeer as a case species (see Web App: Panzacchi and van Moorter 2022a; NINA 2022b) but can be applied to any species, as illustrated in another Prototype Web App for green infrastructures (Panzacchi and van Moorter 2022b; NINA 2022a).

The approach builds on the analysis of species’ occurrence data (GPS positions for more than 400 reindeer throughout the distribution range in Norway) together with a large number of environmental covariates describing vegetation, topography, climate, and infrastructures (e.g., roads, hiking trails, hydropower reservoirs, powerlines, tourist cottages, and private cottages). This multistep approach and all resulting maps for Norway are illustrated in the Web App (Panzacchi and van Moorter 2022a). Data were first analyzed using niche modeling approaches to estimate habitat preferences (or habitat loss; Panzacchi et al. 2015) and permeability to reindeer movements (or fragmentation due to barriers; Panzacchi et al. 2016) for every 100 m pixel in Norway. Although these approaches are widely used to assess habitat loss, the resulting estimates refer to each pixel “in isolation”, irrespective of surrounding areas, and thus ignore a key aspect of ecology: connectivity. We therefore used ConScape (van Moorter et al. 2023; van Moorter 2023) to scale up this classical, pixel-focused approach to a landscape-network perspective. The software uses two input variables (i.e., habitat quality and permeability) and applies advanced network modeling procedures based on the Randomized Shortest Path algorithm (Kivimäki et al. 2014, 2020) to estimate the functionality of the entire landscape.

**Fig. 1:**
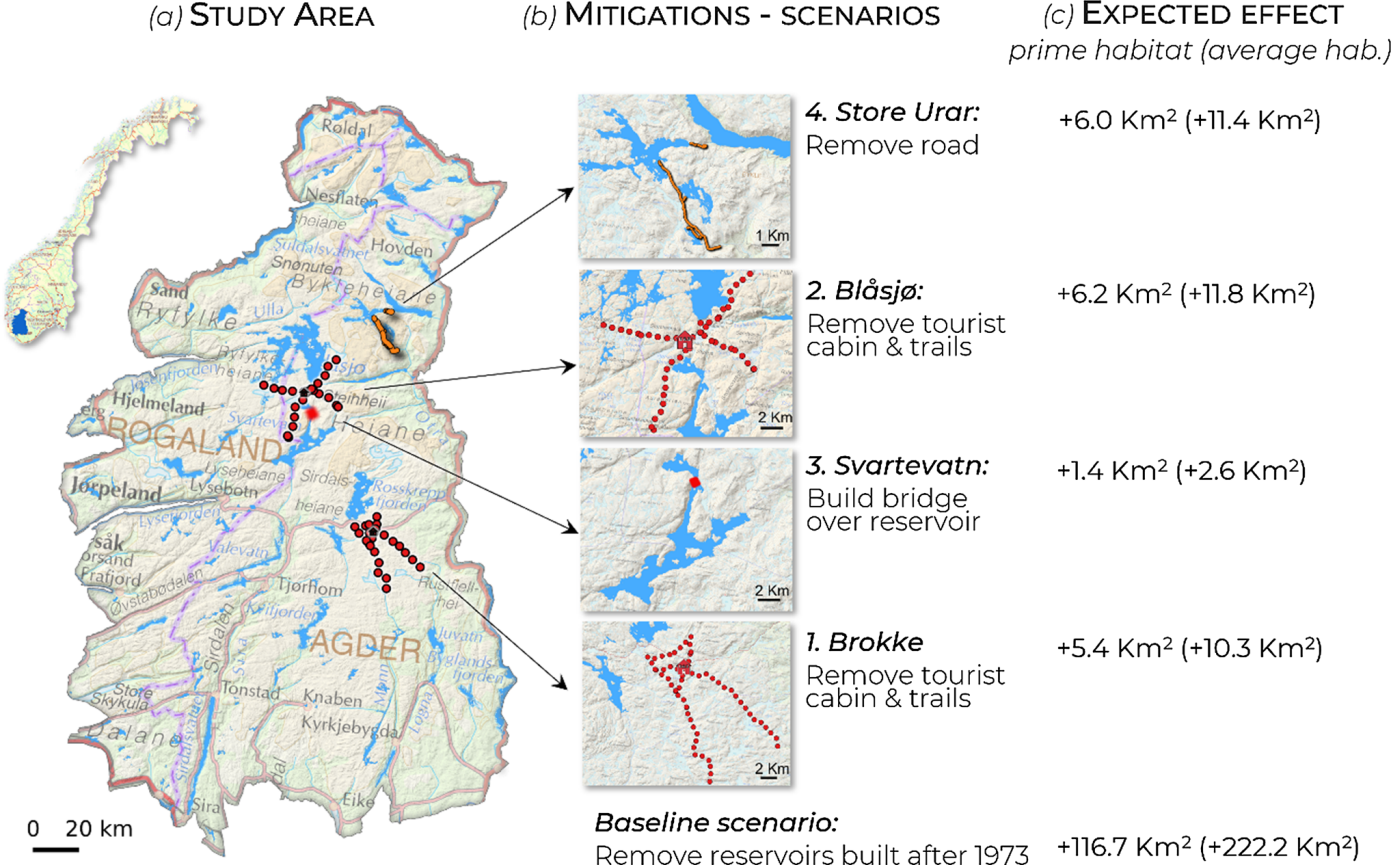
**a** Map of the study area in south Norway (Setesdal Ryfylke wild reindeer management area) highlighting the reservoirs (blue) and an overview of the 4 measures proposed by a consortium of local stakeholders to mitigate or offset the impact of hydropower. **b** Details of each proposed scenario, including a baseline scenario removing all reservoir built after 1973. **c** Results of the scenario analysis, expressed as a km.^2^ gain in functional habitat (Equivalent Connected Habitat) for reindeer. The results are expressed both as the expected gain in prime functional reindeer habitat (0.5% top-quality) and, more realistically, in terms of the gain in average-quality habitat typically used by reindeer (between brackets; see Appendix). The scenarios illustrated here refer to summer, but scenarios for winter (involving the removal of ski trails) have also been tested. For scenario 1, Brokke, we also tested 37 alternative sustainable development options to help managers and stakeholders to re-locate trails and cabins in less important areas for reindeer. All results are given in Panzacchi et al. 2022 and can be viewed in the Web App (Panzacchi and van Moorter 2022a, b)

Here, we apply ConScape to quantify the amount of functional habitat in each pixel of the southernmost wild reindeer management areas, Setesdal Ryfylke (Fig. 1), based on habitat quality and permeability maps for wild reindeer in Norway (Panzacchi et al. 2015, 2016, 2022). The resulting continuous, spatially explicit, pixel-based metric is then synthesized with one value representing the functionality of the entire landscape, expressed as “Equivalent Connected Habitat” (ECH; van Moorter et al. 2023). ECH is useful to quantify functional habitat, to compare habitat loss in before and after land development scenarios, and to compare scenarios of mitigation measures. However, ECH is often expressed in a unit that is difficult to interpret for management purposes. To facilitate its interpretation, we translated ECH into km^2^ of functional habitat lost, and we present all results in two ways (details in the Appendix). First, we indicate the amount of prime reindeer habitat (km^2^, top 0.5% of best habitat) lost or gained and second, we provide a more realistic estimate based on the amount of habitat of average quality, that is typically used by reindeer.

**Fig. 2:**
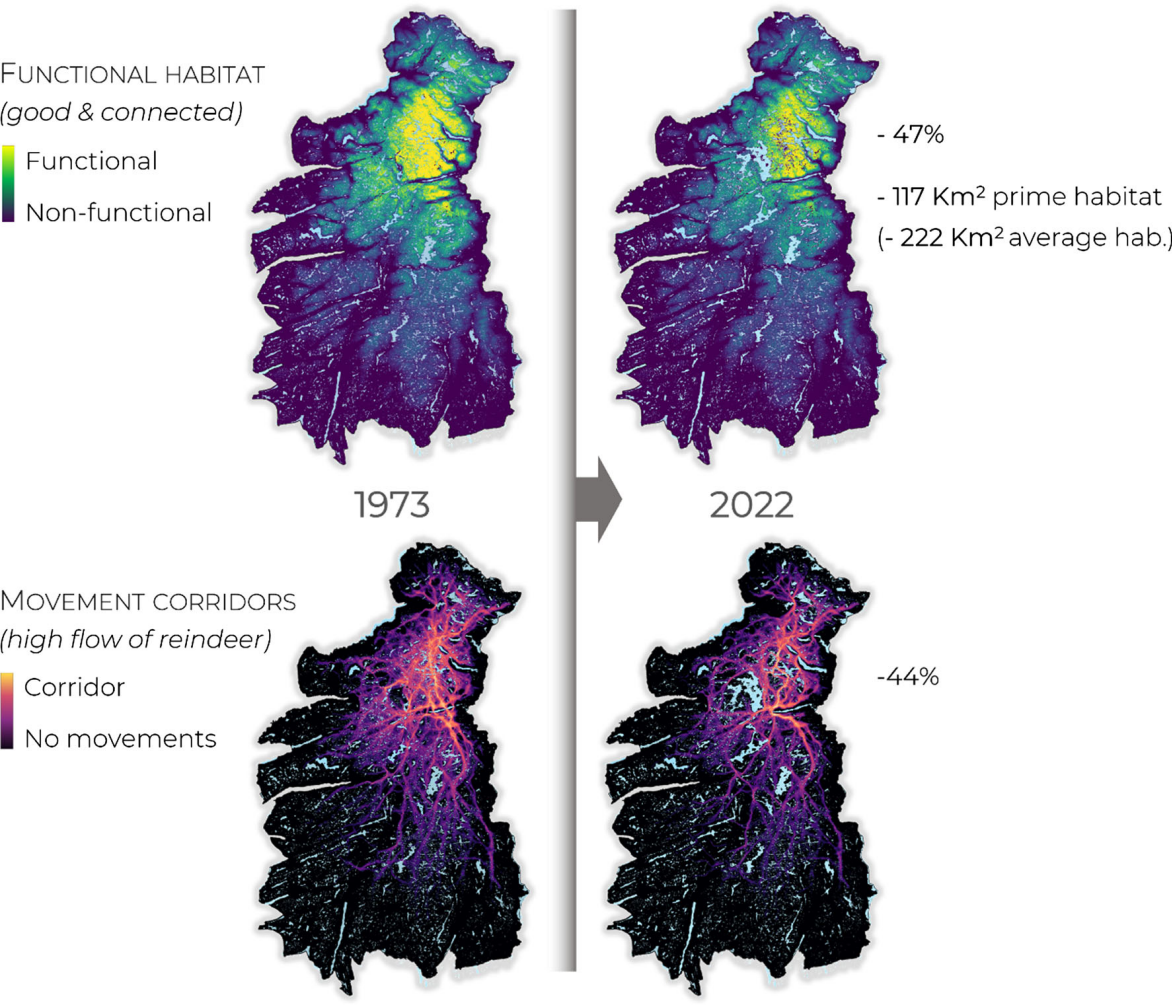
Functional habitat and movement corridors for reindeer in 1973, before the construction of the largest reservoirs (left), and in present times, with one of the largest networks of reservoirs in Europe (right). The reservoirs (light blue) caused habitat loss and fragmentation, and their joint impact is a substantial reduction in the amount of functional habitat (− 47% ECH) and movement possibilities (− 44%). These percentages can be translated into an estimated loss of 117 km^2^ of prime functional reindeer habitat (0.5% top-quality, perfectly connected habitat) or a more realistic estimate of a loss of 222 km^2^ of average-quality habitat, typically used by GPS monitored reindeer (see Appendix). Note that these are conservative estimates of the total impact of hydropower on reindeer (see text)

This differentiation was necessary, as reindeer do not use only “perfect habitat” (i.e., most yellow pixels, Fig. 2), but also use habitat of average functionality (i.e., yellow to green areas). Hence, estimates focusing only on the loss of prime habitat would lead managers to significantly underestimate the actual habitat lost. This was done by quantifying the range of habitat quality values used on average by reindeer, based on their GPS positions. All results consider the cumulative impact of all infrastructure and human activities, given their specific spatial configuration in the landscape.

### Scenario analyses: back-casting and forecasting changes in functional habitat

Once functional habitat and corridors are mapped for the present, the procedure described above can be run iteratively by changing the input layers based on hypothetical or expected changes in the landscape (e.g., removing a tourist cabin and a road) and we can run the procedure again to estimate functional habitat and corridors under different scenarios. In this way, we can estimate ECH for different scenarios of land use changes, or climate changes.

First, we run an unrealistic, hypothetical baseline scenario in which all hydropower reservoirs built after 1973 in Setesdal Ryfylke are removed. This was done using data from Dorber et al. (2018), who used Landsat satellite images to calculate the inundated land area of Norwegian hydropower reservoirs built after 1973. By comparing present-day ECH and 1970 (before the reservoirs were built), we could quantify the functional reindeer habitat lost due to hydropower development. Second, we test a realistic scenario, with mitigation measures, that are currently being considered by management.

In the second scenario, we tested the effect of a set of realistic mitigation measures, that are being considered by management to mitigate the impact of hydropower in wild reindeer areas.

### Participatory processes to identify mitigations and offset measures

A list of 76 mitigation and off-set measures has been suggested and tested in several wild reindeer management areas in Norway, as part of projects on the effect of renewable energy production on reindeer (see details and full list of results in: Panzacchi and van Moorter 2022a). We focus here on Setesdal Ryfylke, the southernmost wild reindeer management areas in Europe, and one of the largest in Norway. The management area extends over 6154 km^2^, and contains 69 hydropower reservoirs, 41 cabins and 3426 km of hiking paths.

To identify a list of realistic mitigation and off-set measures to reduce the impact of hydropower, we engaged in a collaborative process involving a consortium of local reindeer experts and stakeholders including the hydropower industry, the tourist association, and the public administration. The consortium proposed four possible measures (Fig. 1) and anticipated both their efficacy for reindeer and the potential for societal conflict (Strand et al. 2011, 2019). Although all measures aimed at reducing the impact of hydropower on reindeer, only one suggestion addressed the reservoir directly: the construction of a land bridge across a magazine (Scenario 3, Fig. 1). The other interventions suggested represented off-set measures addressing the network of infrastructures in the areas in between reservoirs, which often have become bottlenecks for reindeer movements. These measures include the removal or closure of roads (Scenario 1), and the closure or relocation of hiking or skiing trails and tourist cabins (Scenario 2 and 4). In one case (Scenario 4), where local management is considering removing a major tourist cottage and several trails, we also tested for 37 alternative development scenarios (Panzacchi et al. 2022), to help compensate for the loss by relocating trails and cabins in areas where the impact on reindeer would be smaller. We tested the expected efficacy of the proposed measures in terms of ECH gained as described above.

## Results

### Impact of hydropower reservoirs

**Fig. 3:**
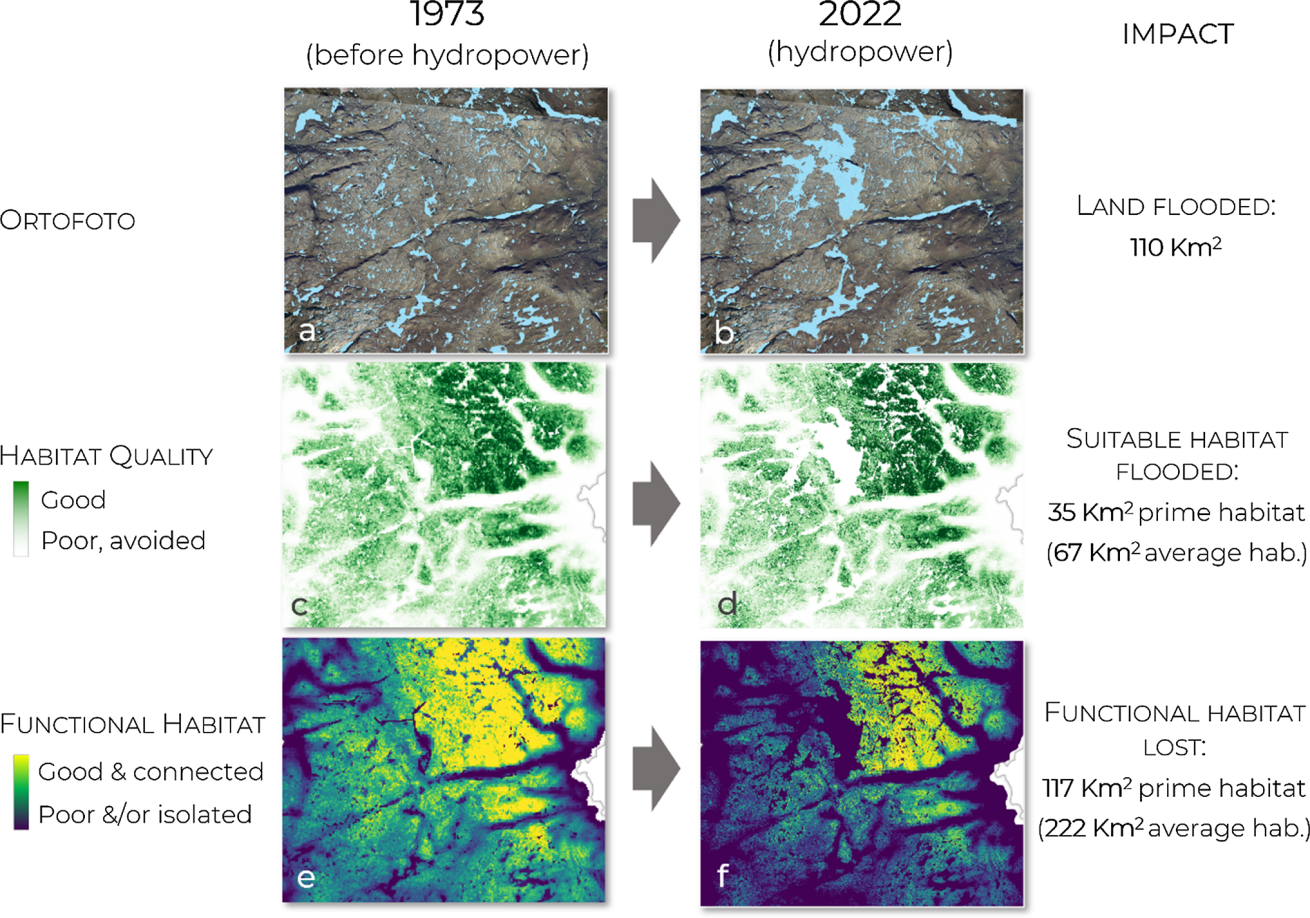
Comparison between 3 increasingly accurate ways to calculate the impact of hydropower development in Setesdal Ryfylke from 1973 (left) to 2022 (right) on reindeer: **a** and **b** amount of land flooded; **c** and **d** amount of suitable habitat flooded—without accounting for connectivity (assessed using Resource Selection Function; Panzacchi et al. 2016); **e** and **f** loss of functional habitat, i.e., simultaneously suitable and well connected (van Moorter et al. 2023). Habitat loss is estimated by Equivalent Connected Habitat and is translated in km^2^ of habitat lost in two ways: focusing on the loss of prime reindeer habitat (0.5% top-quality), and on the loss of average-quality habitat, typically used by reindeer (between brackets; see Appendix). The figures zoom in on the central part of the study area to visualize some of the largest hydropower complex in Europe (Blåsjø), but all estimates refer to the entire Setesdal Ryfylke (Fig. 1; the grey line in c-f delimits the study area)

From 1973 to 2022, 110 km^2^ of land were inundated due to the construction of reservoirs in Setesdal Ryfylke (Fig. 3a, b). However, the area inundated may include both suitable and unsuitable habitat for reindeer. If we consider only the amount of suitable habitat inundated, the loss amounts to 35 km^2^ of prime habitat or, 67 km^2^ of average habitat, typically used by reindeer (Fig. 3c, d). The major problem with both estimates is that they ignore connectivity. This is visualized in a spatially explicit way in Fig. 2, showing changes in functional habitat (areas simultaneously suitable and well connected), and movement corridors (areas through which a high movement flow of reindeer is expected). The construction of hydropower reservoirs caused a 44% loss of connectivity and a 47% decrease in habitat functionality. These values can be translated into a loss of 117 km^2^ of prime, top-quality reindeer habitat, or a loss of 222 km^2^ of average-quality habitat, which is commonly used by reindeer (Fig. 3e, f). Note that these values still underestimate the total impact of hydropower on reindeer because: (i) they refer only to the loss of summer pastures; (ii) they refer only to reservoirs built after 1973; (iii) we did not simulate the removal of hydropower-related infrastructure (e.g., access roads and powerlines); (iv) we present conservative estimates—if we would consider the loss of all areas that could be used randomly by reindeer the value would increase to 530 km^2^. Still, it is substantially larger than the values obtained when ignoring connectivity, as done in most traditional assessments.

### Efficacy of mitigation/offset measures

The expected efficacy of the 4 proposed mitigation and offset measures is presented in Fig. 1c. Although these interventions are very significant from a management and societal perspective, their expected efficacy for reindeer is positive, but minimal if compared to the damage done by the construction of the reservoirs. While 117 km^2^ of prime reindeer habitat were lost due to the construction of the reservoirs, each of the proposed mitigation measures would yield only 1.39 to 6.21 km^2^ of prime habitat. More realistically, while the reservoirs impacted 222 km^2^ of average-quality habitat typically used by reindeer, each of the proposed mitigations would lead to a gain of only 2 to 12 km^2^ of such habitat. The mitigation measure expected to lead to the biggest yield of functional habitat (Fig. 1c, Blåsjø) implies removing tourist infrastructures placed in between several very large reservoirs, in the middle of a corridor that could be used by reindeer to move between suitable pastures in the northern and southern parts of the study area (Fig. 2).

## Discussion

We demonstrate that the cumulative effect of hydropower and associated infrastructure stretches far beyond the reservoir by affecting the entire reindeer ecological network. This shows that striving for SDG 7 can hamper progress on SDG 15 (PEER 2019). More specifically, we estimated that 117 km^2^ of prime functional reindeer habitat, or 222 km^2^ of average-quality habitat used by the species, were lost due to the construction of the reservoirs in Setesdal Ryfylke since 1973. This number could rise to 530 km^2^ if we consider all habitats more than randomly preferred by reindeer. At the same time, our functional habitat loss estimates still represent an underestimation of the actual functional habitat loss for reindeer. First, although we could only simulate the removal of reservoirs constructed after 1973, due to the availability of satellite images (Dorber et al. 2018), the inclusion of reservoirs built before 1973 would further increase the functional habitat loss. Second, we did not simulate the removal of infrastructure associated with hydropower (e.g., access roads and powerlines), as we lacked historical data for infrastructure, although these contributes to the cumulative impact of hydropower. Third, for simplicity, we present estimates of habitat loss only for summer pastures, while the loss of winter and calving habitat should also be integrated, as reindeer are a migratory species, and tend to use different areas in different seasons. Hence, to achieve a sustainable hydropower development, it is not enough to only assess the current habitat loss, as additional mitigation measures are needed.

This confirms that the pure amount of inundated land (110 km^2^), ignoring connectivity, is an oversimplified indicator (Lyytimäki et al. 2020) and that its use can be one explanation for the slow progress towards SDG 15 (PEER 2019). As biodiversity-related SDGs have a positive synergy with almost all SDGs (Blicharska et al. 2019), not reaching SDG 15 further involves the risk of triggering complex chains of cascading impacts across all SDGs (PEER 2019). Thus, sustainable hydropower production can only be achieved if, in addition to the positive synergies, biodiversity trade-off risks are considered a fundamental layer in the decision. Hence, neither technical feasibility, electricity price (Gernaat et al. 2017) or greenhouse gas emissions of hydropower electricity production alone (Hertwich 2013) should be the basis for sustainable hydropower development decisions.

Compensating entirely for the significant loss of functional habitat due to hydropower would be utopian. Hence, we estimated the expected gain in functional habitat associated with a range of more realistic mitigation or offset measures suggested by local stakeholders. Notwithstanding the significant societal engagement, and the significant effort and cost that would be required to implement those measures, we estimated that the functional habitat that would be gained by such measures would be minimal, compared to the total loss caused by hydropower development (1 to 6 km^2^ of prime habitat). Nevertheless, our study shows that involvement of local stakeholders can lead to the identification of feasible and socially acceptable mitigation measures, which are key to identify sustainable solutions for land planning related to wind and hydropower projects (IEA 2021).

At the same time, the here assessed offset measures (i.e., removal of tourist cabins, hiking trails and roads) can also cause unintended trade-off risks with other SDGs. Specifically, these measures could a trade-off risk with SDG 8.9, which aims to *“implement policies to promote sustainable tourism that creates jobs and promotes local culture"* (United Nations 2015), or with SDG 12.b, which aims to “*develop and implement tools to monitor sustainable development impacts for sustainable tourism which creates jobs, promotes local culture and products*” (United Nations 2015), or SDG 3 *“Good health and well-being”* in general. When it comes to roads, Ibisch et al. (2016) identified, that despite the positive synergies with SDG 15, conservation of roadless areas has a negative trade-off with nine other SDGs (i.e., 1, 2, 3, 4, 7, 8, 9 and 10). This confirms that it is best to avoid major impacts in the first place (Kiesecker et al. 2010), by following the “mitigation hierarchy”, where scientists and societal actors jointly collaborate from an early stage to plan the most sustainable land development alternatives. The simulation tool that we developed can be also used for this purpose. If offset measures are needed, it is important that sustainable hydropower development accounts for their trade-off risks as they can affect economic, political, and societal interests far beyond the hydropower industry itself. While being beyond the scope of this study, one possible starting point could be the approach from Nilsson et al. (2016), rating positive synergies and trade-offs from -3 to 3. This scale could then be used to identify the offset measures that are best from a holistic SDG perspective.

In addition, it is questionable if it is sufficient to only consider bottom-up mitigation or offset measures (proposed by local stakeholders), or if top-down approaches (governmental level) would be needed. In parallel, it has to be considered that in contrast to minimum flow requirements, the measures assessed here do not interfere with the electricity production itself (Köhler and Ruud 2019). Consequently, here a range of societal actors are bearing the burden of mitigating the effect of hydropower, thus raising the question of how the economic costs of the offset measures should be distributed.

Although the case study we presented refers to south Norway, the approach is also being used for wild and semi domestic reindeer in Norway and Sweden. Furthermore, it is also being used to identify functional, connected habitat to be prioritized for conservation or restoration in a multi-species context (see prototype Web App: Panzacchi and van Moorter 2022b). Some of the results presented here are currently being considered to identify adequate mitigation measures to support the relicensing process for hydropower production in Norway, and to support sustainable management strategies for reindeer. Furthermore, the theoretical and methodological framework we propose is applicable to any species and country, and ConScape (van Moorter et al. 2023; van Moorter 2023) can be used to promote sustainable land management from the local scale to a regional, national and international context, as it allows to quantify cumulative impacts caused by a range of infrastructures. Future research should focus on establishing a precise link between the concept of habitat functionality (and ECH) and population dynamics. Although this concept has been studied for decades (Boyce et al. 1999; Boyce et al. 2016), and we do know that cumulative impacts lead to a reduction in carrying capacity (van Moorter et al. 2020), the conversion between habitat units and population viability still remains a Holy Grail in ecological studies.

Overall, our study highlights the need and the urgency to consider quantitative, network-based estimates of functional connectivity as an indicator to assess the amount of suitable habitat accessible to species, and the cumulative impacts of infrastructure. Only with such indicator we can avoid a major underestimation of impacts in several types of environmental reporting, sustainability assessments and in Environmental Impact Assessment (EIA) studies—including for renewable energy. As more accurate and science-driven indicators are needed to bend the curve of biodiversity decline (Mace et al. 2018), cumulative impact studies and studies on “connectivity conservation” are currently proliferating, and their policy-related applications are gaining traction in international and national policies. For instance, connectivity is a key component of the *EU 2020 Biodiversity Strategy* (Maes et al. 2016) and of the Convention on Biological Diversity—*Aichi Target 11* (CBD 2020). While several indicators are currently being considered to monitor ecological connectivity for the post-2020 global biodiversity framework, no ideal solution seems to have been identified yet. Consequently, currently in several studies connectivity is often either overlooked, or accounted for in simplistic ways. However, the estimates of functional habitat loss can be used as land occupation value for hydropower in Life Cycle Assessment (LCA) (ISO 2006), one of the tools identified by Liu et al. (2018) to assess nexus approaches between SDGs. As LCA is, for example, implemented by the EU in policies (Sala et al. 2021), our values can support decision-making in the renewable energy sector. This further supports the idea that the approach proposed here, and the software ConScape, may help to achieve SDG 15.9, which aims to *“integrate ecosystem and biodiversity values into national and local planning, development processes and poverty reduction strategies, and accounts”* (UN 2015).

Note that the approach presented in this study builds upon and represents a generalization of existing indicators suggested to measure area functionality for Aichi Target 11 (i.e., ProtConn; Saura et al. 2017, 2018). The ProtConn indicator assesses connectivity using the cost of a single (i.e., the Least-Cost) path for protected areas. As it was developed to assess connectivity of protected areas, it ignores the role of non-protected areas to biodiversity. Our indicator, therefore, extends ProtConn by considering: *(a)* habitat as continuous concept depending upon the environmental characteristics of each pixel; *(b)* the contribution of all paths weighted by their likelihood and cost using the Randomized Shortest Path framework (Saerens et al. 2009). Hence, the indicator we present here has a potential to advance the current state of the art in policy and decision-making at several levels, and to be of use for connectivity assessment and sustainable land planning beyond the focal species of this study, reindeer, and beyond hydropower (Panzacchi and van Moorter 2022b), e.g., tourism, transportation, and wind power. This is especially relevant as SDG 7 cannot be exclusively achieved through hydropower but requires combining different renewable energy sources (Bogdanov et al. 2019). Other renewable energy sources like wind power are also known to cause habitat loss and fragmentation for a range of species, including *Rangifer* (Johnson et al. 2015; Skarin et al. 2015, 2018; Marques et al. 2019).

## Conclusion

To achieve the 17 sustainability goals, we need to make sure that we not only focus on positive synergies, but that we also account for negative trade-off risks. Here we showed a major trade-off between renewable energy production and conservation of species’ habitats, that needs to be acknowledged. At the same time, we need to avoid the use of oversimplified indicators to assess trade-off risks. Based on our results, we recommend using indicators of functional habitat loss (i.e., accounting for cumulative impacts on the functional connectivity of ecological networks) as indicator for anthropogenic impacts of land use changes, to avoid major underestimations in environmental impact assessment studies, and in policy reports for biodiversity monitoring.

Furthermore, we showed that participatory processes involving local stakeholders are crucial to identify feasible and socially acceptable measures to mitigate habitat loss. However, we also showed that the habitat gained by such measures is minimal, compared to the habitat lost by hydropower development, that likely will never be fully recuperated. Therefore, we recommend following the mitigation hierarchy (prevent, mitigate, restore, offset, compensate) from the early planning stages of all renewable energy development projects. In other words, adequate and science-driven planning can help to prevent major impacts and SDG trade-off risks in the first place, and simulations can be a valuable preventive tool to support this process. Last, our results show that offset measures can trigger a cascade of new wicked trade-off risks with other SDGs, which also have to be considered. The ConScape software can be a valuable decision support tool to quantify trade-off risks between biodiversity conservation and sustainable development.

## Supplementary Information

This supplementary material has not been peer reviewed.

Supplementary file1 (PDF 111 kb)
