# Supplementary material for: New indicator of habitat functionality reveals high risk of underestimating trade-offs among sustainable development goals: The case of wild reindeer and hydropower
Source: Ambio. 2023 Feb 9;52(4):757–68. doi: 10.1007/s13280-022-01824-x (PMC9989093; doi:10.1007/s13280-022-01824-x)
Supplement: Supplementary file 1 — Supplementary file1 (PDF 111 kb) [file 13280_2022_1824_MOESM1_ESM.pdf]

Ambio

Electronic Supplementary Material
This supplementary material has not been peer reviewed.

Title: **New indicator of habitat functionality reveals high risk of underestimating trade-offs among sustainable development goals - The case of wild reindeer and hydropower**

Authors: Martin Dorber, Manuela Panzacchi, Olav Strand, Bram van Moorter

Appendix I

Quantifying changes in functional habitat using Equivalent Connected Habitat

Authorities, managers and land planners need to know how many square kilometers of "useful" or "functional" reindeer pastures have been lost due to hydropower development in order to plan adequate compensations, or how many km^2 could be gained by implementing different measures to mitigate the damage done. In this paper we addressed this need by quantifying and mapping functional habitat (high-quality, well-connected habitat) for each 100 x 100 m pixel in the landscape, in a continuous way (van Moorter et al. 2023). We then synthesized this spatially explicit, pixel-based metric with one value representing the functionality of the entire landscape, expressed as "Equivalent Connected Habitat" (ECH; van Moorter et al. 2023). ECH is a useful currency to quantify functional habitat, to calculate habitat loss by comparing before-after scenarios of land development, and by comparing scenarios of mitigation measures. However, the ECH is a volume, and not an area, which makes it challenging to interpret in terms of km^2 lost or gained, and directly meet managers' needs. To meet this challenge, here we describe two ways to translate ECH into km^2 of reindeer pastures, to facilitate interpretation in an applied land management context.

Equivalent Connected Habitat - ECH

The ECH builds on the concepts of habitat quality and connectivity, both of which are measured on a continuous scale. ECH is an extension of the Equivalent Connected Area, ECA, earlier developed by Saura et al. (2011) for habitat patches, to allow for continuous variations in habitat quality. For a given landscape, the ECA (Saura et al. 2011) quantifies the amount of high-quality habitat, in m^2, if it were to occur in one single patch (i.e., if it was all well connected). The limitation of the ECA is that it only works for a patch representation of habitat, i.e., each landscape unit ("pixel") can either be habitat or not. This simplified representation obviously fails to account for the continuous nature of habitat quality. The ECH is therefore an extension of the ECA developed to allow for continuous variations in habitat quality. Unfortunately, this generalization comes at a cost, as the ECH is no longer an area (measured in m^2), but it is a volume, whose dimensions are the coordinates X, Y, and "habitat quality". Hence, the measurement unit of ECH is "m^2 habitat" (habitat is here a short for habitat quality). However, "habitat" has no recognized measurement unit or scale, which makes it a difficult metric to use in an applied context. Although no approach can be fully satisfactory in "squeezing" a 3D volume into a 2D area, as the process necessarily implies loss of information, here we describe two ways to translate ECH into km^2 of reindeer pastures, to facilitate interpretation in an applied land management context. First, we present ECH in units of prime reindeer habitat (top 0.5% best habitat) and, second, we present ECH in units of habitat typically (i.e., on average) used by reindeer. Both metrics are presented to help interpretation of our results.

1) Measuring habitat in units of "prime reindeer habitat"

Habitat quality was estimated using a resource selection function (RSF; Fig 3 c, d; Panzacchi et al. 2015), which is commonly used for this purpose. However, the RSF results from a linear model using a log-link $[0,\infty]$. Hence, the measurement scale of the RSF is not an absolute, but a relative one, or a ratio (Fieberg et al. 2021). We rescaled the RSF values to make 1 correspond to the 99.5% percentile (and truncated values above 1) – the RSF (or habitat quality) $\in [0,1]$. Therefore, the unit (i.e., value 1) of habitat quality corresponds to the 0.5% best pixels in the study area, or "prime habitat". Thus, an ECH of 1 m^2 *habitat* equals 1 m^2 of prime and perfectly connected habitat. Note that as all pixels have RSF values ≤ 1, the area of m^2 *habitat* can never surpass the area in m^2. In other words, ECH can never be larger than the size of the available area. The representation of ECH as "prime functional habitat" is reasonable, intuitive, and it is useful for comparing changes in functional habitat under different

scenarios. However, focusing on prime, top-quality habitat necessarily leads the reader to severely underestimate the amount of area that can actually be used by reindeer.

2) Measuring habitat in units of “average habitat used by reindeer”

In practice, reindeer use habitat of varying quality, and do not limit themselves only to the top 0.5% of the best habitat. In fact, only 4% of GPS locations fall in the category of prime reindeer habitat. Hence, prime habitat is highly preferred by reindeer, but nevertheless most of the time reindeer use habitat of subprime quality. Therefore, expressing the useful habitat for a species that is lost due to infrastructure development in units of prime habitat may be strongly misleading, especially in an applied context. It may therefore be more appropriate to use a different measurement unit for habitat, and focus on the habitat typically used by reindeer, rather than only the 0.5% top-quality habitat.

Here we focused on the quality of the habitat actually used by the animals (as measured by GPS tracking data), and we scaled the ECH accordingly. Specifically, we calculated the average RSF values used by the animals, which is 0.53. Then, by dividing our ECH values in ‘prime habitat units’ by 0.53, we rescaled the ECH to units of average used habitat. Thus, an ECH of 1 m^2 *habitat* would now correspond to 1 m^2 of used habitat. Note however that in doing so pixel values may be ≥ 1 (in our case all pixel values > 0.53 would become > 1 in these new habitat quality units), and therefore the area of m^2 *habitat* (derived from ECH) could be larger than the area in m^2. This might lead to the somewhat counter intuitive result that the habitat lost (from the RSF, i.e., without accounting for fragmentation) in m^2*habitat* due to a reservoir may be larger than the area flooded by the reservoir itself in m^2.

Conclusion

The ECH is a very useful metric to quantify loss of functional habitat (high quality and well connected) and compare the effect of scenarios of landscape changes. However, as ECH is measured in “m^2 habitat quality”, due to the absence of a recognized metric for habitat quality, there is no perfect solution to express ECH in units easy to communicate for administrative and land management purposes. We therefore opted to describe habitat loss first and foremost in terms of loss of prime habitat, which is a comprehensive, comparable and sound metric. To avoid the risk that managers could underestimate, also significantly, the amount of habitat lost for the species, we also chose to indicate the loss of ECH in units of average habitat used typically by reindeer, which provides a more realistic estimate of the impact of infrastructures.

References

Fieberg, J, Signer, J, Smith, B, Avgar, T. A ‘How to’ guide for interpreting parameters in habitat-selection analyses. J Anim Ecol. 2021; 90: 1027– 1043.

Panzacchi, M., van Moorter, B., Strand, O., Loe, L. E., and Reimers, E. (2015). Searching for the fundamental niche using individual-based habitat selection modelling across populations. Ecography, 38(7):659–669

Saura, S., Estreguil, C., Mouton, C., and Rodríguez-Freire, M. (2011). Network analysis to assess landscape connectivity trends: application to european forests (1990–2000). Ecological Indicators, 11(2):407–416

Van Moorter, B., Kivimäki, I., Noack, A., Devooght, R., Panzacchi, M., Hall, K.R., Leleux, P. and Saerens, M. (2023), Accelerating advances in landscape connectivity modeling with the ConScape library. Methods in Ecology and Evolution, 14:143-145
